# Supplementary material for: The Systems Biology Research Tool: evolvable open-source software
Source: BMC Syst Biol. 2008 Jun 29;2:55. doi: 10.1186/1752-0509-2-55 (PMC2446383; doi:10.1186/1752-0509-2-55)
Supplement: Additional file 1 — SBRT Archive. An archive of the current version of the Systems Biology Research Tool. [file 1752-0509-2-55-S1.zip › sbrt-1.4.0/doc/developers_guide/api/sbrt/shell/io/fba/FluxIntervalInputFile.html]

FluxIntervalInputFile


|  |  |  |
| --- | --- | --- |
| **Overview** \| **Package** \| **Class** \| **Use** \| **Tree** \| **Deprecated** \| **Index** \| **Help** | |  |
| **PREV CLASS**   **NEXT CLASS** | **FRAMES**    **NO FRAMES**     **All Classes** |  |
| SUMMARY: NESTED \| FIELD \| CONSTR \| METHOD | DETAIL: FIELD \| CONSTR \| METHOD |  |


---


## sbrt.shell.io.fba Class FluxIntervalInputFile

```
java.lang.Object
  sbrt.shell.io.fba.FluxIntervalInputFile
```

**All Implemented Interfaces:**: java.lang.Iterable<FluxConstraints>, InputFile<FluxConstraints>, SbrtFile

---

``` public class FluxIntervalInputFile extends java.lang.Object implements InputFile<FluxConstraints> ```

This class is used to read flux interval input files.

**Author:**
:   This class was written and documented by Jeremiah Wright while in the
    Wagner lab.

---

**Constructor Summary**

`FluxIntervalInputFile(java.lang.String fileName,
FileFormat fileFormat,
java.util.Set<LinearComb<java.lang.String>> requiredVariables,
MapFormat<LinearComb<java.lang.String>,Interval> mapFormat)`
  
          Constructs a new flux-interval input file.


| **Method Summary** | |
| --- | --- |
| `void` | `close()`             Closes this file. |
| `static FluxConstraints` | `convertConstraints(java.util.Map<LinearComb<java.lang.String>,Interval> intervals)`             Converts the provided intervals into a `FluxConstraints` object. |
| `static java.util.Set<LinearComb<java.lang.String>>` | `convertVariables(java.util.Set<java.lang.String> rxnNames)`             Converts a set of reaction names to a set of single-term linear combinations. |
| `java.lang.String` | `getCanonicalName()`             Returns the canonical name of this file. |
| `java.lang.String` | `getOriginalName()`             Returns the name of this file. |
| `boolean` | `isClosed()`             Indicates if this file has already been closed. |
| `java.util.Iterator<FluxConstraints>` | `iterator()`             Returns an iterator over the flux intervals contained in this file. |

**Methods inherited from class java.lang.Object**

`clone, equals, finalize, getClass, hashCode, notify, notifyAll, toString, wait, wait, wait`

**Constructor Detail**

### FluxIntervalInputFile

```
public FluxIntervalInputFile(java.lang.String fileName,
                             FileFormat fileFormat,
                             java.util.Set<LinearComb<java.lang.String>> requiredVariables,
                             MapFormat<LinearComb<java.lang.String>,Interval> mapFormat)
```

:   Constructs a new flux-interval input file.

    **Parameters:**: `fileName` - the name of the file to be read.: `fileFormat` - the format of the file to be read.: `requiredVariables` - the variables that must be contained in the file to be read.: `mapFormat` - the format of data strings in the file to be read.


**Method Detail**

### close

```
public void close()
```

:   Closes this file. If this file is already closed, then invoking this
    method has no effect.

    :   **Specified by:**: `close` in interface `SbrtFile`

---


### getOriginalName

```
public final java.lang.String getOriginalName()
```

:   Returns the name of this file.

    :   **Specified by:**: `getOriginalName` in interface `SbrtFile`
    :   **Returns:**: the name of this file.

---


### getCanonicalName

```
public final java.lang.String getCanonicalName()
```

:   Returns the canonical name of this file.

    :   **Specified by:**: `getCanonicalName` in interface `SbrtFile`
    :   **Returns:**: the canonical name of this file. **See Also:**: `File.getCanonicalPath()`

---


### isClosed

```
public boolean isClosed()
```

:   Indicates if this file has already been closed.

    :   **Specified by:**: `isClosed` in interface `SbrtFile`
    :   **Returns:**: `true` if this file is closed; `false` othewise.

---


### iterator

```
public java.util.Iterator<FluxConstraints> iterator()
```

:   Returns an iterator over the flux intervals contained in this file.

    :   **Specified by:**: `iterator` in interface `java.lang.Iterable<FluxConstraints>`
    :   **Returns:**: an iterator over the flux intervals contained in this file.

---


### convertVariables

```
public static java.util.Set<LinearComb<java.lang.String>> convertVariables(java.util.Set<java.lang.String> rxnNames)
```

:   Converts a set of reaction names to a set of single-term linear
    combinations.

    :   **Parameters:**: `rxnNames` - the reaction names to be used for constructing single-term linear combinations. **Returns:**: a set of single-term linear combinations corresponding to the provided reaction names.

---


### convertConstraints

```
public static FluxConstraints convertConstraints(java.util.Map<LinearComb<java.lang.String>,Interval> intervals)
```

:   Converts the provided intervals into a `FluxConstraints` object. If
    a provided linear combination contains a single term, with coefficient
    equal to 1, and constant equal to 0, the interval will be applied to the
    variable itself (not the linear combination), which is retrievable using
    `FluxConstraints.getConstraint(String)` and
    `FluxConstraints.getFluxConstraints()`.

    :   **Parameters:**: `intervals` - reaction name expressions mapped to their corresponding interval. **Returns:**: a `FluxConstraints` object corresponding to the provided input.


---


|  |  |  |
| --- | --- | --- |
| **Overview** \| **Package** \| **Class** \| **Use** \| **Tree** \| **Deprecated** \| **Index** \| **Help** | |  |
| **PREV CLASS**   **NEXT CLASS** | **FRAMES**    **NO FRAMES**     **All Classes** |  |
| SUMMARY: NESTED \| FIELD \| CONSTR \| METHOD | DETAIL: FIELD \| CONSTR \| METHOD |  |


---
